# Impact of Ocean Acidification on Energy Metabolism of Oyster, *Crassostrea gigas*—Changes in Metabolic Pathways and Thermal Response

**DOI:** 10.3390/md8082318

**Published:** 2010-08-11

**Authors:** Gisela Lannig, Silke Eilers, Hans O. Pörtner, Inna M. Sokolova, Christian Bock

**Affiliations:** 1 Alfred Wegener Institute for Polar and Marine Research in the Hermann von Helmholtz Association of National Research Centres e.V. (HGF), Am Handelshafen 12, 27570 Bremerhaven, Germany; E-Mails: Silke.Eilers@gmx.net (S.E.); Hans.Poertner@awi.de (H.O.P.); Christian.Bock@awi.de (C.B.); 2 Department of Biology, University of North Carolina at Charlotte, 9201 University City Blvd., Charlotte NC, 28223, USA; E-Mail: ISokolov@uncc.edu (I.M.S.)

**Keywords:** ^1^H-NMR spectroscopy, acute warming, long-term hypercapnia, acid-base status, metabolic profiling, Na^+^/K^+^-ATPase

## Abstract

Climate change with increasing temperature and ocean acidification (OA) poses risks for marine ecosystems. According to Pörtner and Farrell [1], synergistic effects of elevated temperature and CO_2_-induced OA on energy metabolism will narrow the thermal tolerance window of marine ectothermal animals. To test this hypothesis, we investigated the effect of an acute temperature rise on energy metabolism of the oyster, *Crassostrea gigas* chronically exposed to elevated CO_2_ levels (partial pressure of CO_2_ in the seawater ~0.15 kPa, seawater pH ~ 7.7). Within one month of incubation at elevated *P*co_2_ and 15 °C hemolymph pH fell (pH_e_ = 7.1 ± 0.2 (CO_2_-group) *vs.* 7.6 ± 0.1 (control)) and *P*_e_co_2_ values in hemolymph increased (0.5 ± 0.2 kPa (CO_2_-group) *vs.* 0.2 ± 0.04 kPa (control)). Slightly but significantly elevated bicarbonate concentrations in the hemolymph of CO_2_-incubated oysters ([HCO^−^ _3_]_e_ = 1.8 ± 0.3 mM (CO_2_-group) *vs.* 1.3 ± 0.1 mM (control)) indicate only minimal regulation of extracellular acid-base status. At the acclimation temperature of 15 °C the OA-induced decrease in pH_e_ did not lead to metabolic depression in oysters as standard metabolism rates (SMR) of CO_2_-exposed oysters were similar to controls. Upon acute warming SMR rose in both groups, but displayed a stronger increase in the CO_2_-incubated group. Investigation in isolated gill cells revealed a similar temperaturedependence of respiration between groups. Furthermore, the fraction of cellular energy demand for ion regulation via Na^+^/K^+^-ATPase was not affected by chronic hypercapnia or temperature. Metabolic profiling using ^1^H-NMR spectroscopy revealed substantial changes in some tissues following OA exposure at 15 °C. In mantle tissue alanine and ATP levels decreased significantly whereas an increase in succinate levels was observed in gill tissue. These findings suggest shifts in metabolic pathways following OA-exposure. Our study confirms that OA affects energy metabolism in oysters and suggests that climate change may affect populations of sessile coastal invertebrates such as mollusks.

## 1. Introduction

Coastal zones are ecologically and economically important and are among those areas that will be strongly affected by global climate change. Increasing atmospheric CO_2_ concentrations lead to increasing mean temperatures and a higher frequency of thermal extremes as well as to ocean acidification (OA). These environmental changes pose risks for marine ectothermal organisms that are not yet fully understood [2–4]. Current ecosystem changes are largely driven by temperature, whereas ecosystem impacts of elevated CO_2_ are expected, but still have to be demonstrated for moderate oceanic *P*co_2_ levels that are predicted to occur over the next 100 years. Previous studies indicate that OA, “global warming’s evil twin” (Richard Feely cited by [5]) will have mainly adverse consequences for many calcifying and non-calcifying organisms, and may result in changes to biodiversity, trophic interactions, and other ecosystem processes [4,6–11]. Marine organisms are affected by OA in several, mainly negative ways as shown by disturbances of acid-base regulation, respiration, energy turnover and metabolism, as well as a reduction in growth rates, reproductive success and calcification, and sensory abilities [12–20]. Depending on future CO_2_ emission scenarios, predicted average surface seawater pH varies between 7.7 and 8.0 [21]. Deviation of environmental factors from the evolutionary optimum for a given species may result in deleterious impacts on energy homeostasis. Sub-optimal conditions create metabolic energy demands that may exceed energy supplied from food and/or accrued in somatic energy resources, as well as overwhelm the capacity of systemic functions (ventilation and circulation) and the cellular metabolic machinery to provide enough adenosine triphosphate (ATP) to sustain routine metabolism. An organism may compensate for elevated energy demand during the moderate stress by increasing energy intake and assimilation, and/or by elevated metabolic flux to cover ATP demand. However, during extreme stress exposures such compensation may be incomplete or impossible, and an organism can enter a metabolically depressed state to conserve energy and to extend the survival time until the conditions return to the optimum [22]. In either case, a disturbance of energy homeostasis will result in a limitation of aerobic scope of an organism, which in turn reduces the thermal tolerance limits of aquatic ectotherms [23]. Indeed, temperature-induced hypoxemia, transition to anaerobic energy production and oxidative stress were shown to play a critical role in determining the thermal tolerance limits and survival at elevated temperatures in aquatic ectotherms [24–29].

Recently, the concept of oxygen- and capacity-limited thermal tolerance of aquatic ectotherms [23] was extended to incorporate CO_2_-driven effects predicting that the acute CO_2_ stress will increase sensitivity of an organism to temperature change [1,4]. Indeed, hypercapnia caused a reduction in hemolymph O_2_ partial pressure (*P*_e_o_2_) and narrowed the thermal tolerance window by a downward shift of the upper critical temperature in crabs, *Cancer pagurus* (1 kPa *P*co_2_, [30]) and *Hyas araneus* (0.3 kPa *P*co_2_, [31]). Michaelidis *et al.* [32] found no differences in *P*_e_O_2_ levels between control and hypercapnia-exposed mussels, *Mytilus galloprovincialis* (0.6 kPa *P*co_2_) when measured at the acclimation temperature. Following OA exposure extracellular pH (pH_e_) dropped by 0.2 units and the standard metabolic rate (SMR) of the mussels decreased by about 60% indicating metabolic depression [32]. Metabolic depression acts as a time-limited adaptation strategy to survive unfavourable conditions such as hypercapnia [22]. Long-term performance and thus fitness is key to survival and success of a species. As summarized by Pörtner *et al.* [15], key physiological processes that are involved in setting the sensitivity to ocean acidification are the regulation of the organisms’ cellular acid-base and ion status and the respective feed back loops on other processes that are associated with individual performance. Regulation of pH_e_ is thought to “be the first line of defense against hypercapnia induced disturbances of metabolic and tissue functioning” (p. 210) emphasizing a key role of pH_e_ in metabolic depression [33].

In contrast to vertebrates, most invertebrates exhibit a low capacity for acid-base regulation such that the changes in acid-base and ion status may directly interfere with the organism’s performance [3,15,34,35]. Thus, the impact of future CO_2_ concentrations is expected to be strong in invertebrates that are weak acid-base regulators and are unable to compensate the OA-induced shift in extracellular pH. Among this group, calcifying organisms may be particularly vulnerable to OA, because in addition to the acid-base disturbances they can experience disturbances in biomineralization needed for production of CaCO_3_ exo- and endoskeletons [32,36–39]. As shown by Gazeau *et al.* [40] and Ries *et al.* [41], acute exposure to OA impairs the calcification of benthic mollusks already at calcium carbonate saturation values above 1 suggesting a decrease by 25% and 10% in mussel and oyster calcification by the end of the century with predicted *P*co_2_ levels of ~740 ppmv. In the mussel *M. galloprovincialis* long-term hypercapnia (seawater pH 7.3, ~0.6 kPa *P*co_2_) caused a reduction in hemolymph pH which was partly compensated by increased [HCO_3_ ^−^]_e_ [32]. The increase in bicarbonate levels was likely linked to shell dissolution as indicated by increased extracellular [Ca^2+^] [32]. The drop in pH_e_ from 7.55 to 7.36 correlated with a reduction in metabolic rate and was suggested to be responsible for the observed growth rate reduction [32]. In *Mytilus edulis* chronically exposed to various CO_2_ concentrations (seawater pH ranged from 6.7 to 8.1) growth increments were reduced when seawater pH fell below 7.4 [42].

In contrast to the bivalves, hypercapnia (0.4–0.6 kPa *P*co_2_, seawater pH of 7.1–7.2) did not depress metabolic rate and growth performance in the cephalopod, *Sepia officinalis*. Extracellular acidosis was compensated for by the accumulation of bicarbonate to a larger degree than in the bivalves but compensation remained incomplete [43]. These data indicate that CO_2_ responses and thus tolerance thresholds may vary between animals depending on taxonomic group, acclimation history and degree of the hypercapnic stress.

Oysters are a major group of calcifiers in estuaries and coastal zones that may be strongly affected by OA and climate change. Estuarine and coastal organisms are regularly exposed to variable temperature and pH values in their environment and thus may be less sensitive to acidification and global warming than their open-ocean counterparts that live in more stable thermal and pH environments [42]. However, estuarine and coastal areas are likely impacted the most in terms of the degrees and rates of projected warming and seawater acidification trends. In combination with lower salinity and thus depressed CaCO_3_ saturation state these trends may strongly affect ectothermic calcifiers such as oysters [44]. We hypothesized that chronic OA exposure will affect energy metabolism in oysters and will have negative consequences on their temperature tolerance. To test this hypothesis we determined the impact of long-term OA/hypercapnia on temperature-dependent metabolism by analyzing the acid-base and metabolic status of the Pacific oyster, *Crassostrea gigas* under conditions simulating a future scenario in which CO_2_ levels stabilize at ~0.1 kPa *P*co_2_ and water pH decreases by ~0.5 units [45]. We show that OA exposure leads to a disturbance of acid-base status and changes in the steady-state levels of metabolic intermediates and induces an increase in basal maintenance costs at elevated temperatures. These disturbances of energy metabolism indicate that performance and survival of estuarine invertebrates can be affected by even moderate OA scenarios supporting the recommendation by Turley *et al.* [46].

## 2. Results and Discussion

During the first days of CO_2_ incubation oysters *C. gigas* showed delayed behavioral defense responses (e.g., were slow to close or did not close their shells in response to a touch; data not shown). However, the behavioural responses normalized during long-term CO_2_ exposure (up to 55 days), and only one oyster out of 23 animals died after 22 days of CO_2_ exposure, which equals a 4.3% mortality rate. No mortality was observed in the control group. Chronic hypercapnia resulted in significant changes in hemolymph parameters of oysters (Table 1). CO_2_-exposed oysters showed elevated *P*_e_co_2_ accompanied by lower hemolymph pH (decrease in pH_e_ by ~0.5 units) compared to controls. A small but significant increase in [HCO^−^ _3_]_e_ was also observed in oysters following chronic hypercapnia. Chronic hypercapnia also affected concentrations of [Na^+^]_e_, [K^+^]_e_ and [Ca^2+^]_e_ in oyster hemolymph (Table 1). Hemolymph oxygenation was slightly affected by long-term hypercapnia with an approximately 20% reduction in hemolymph *P*o_2_ in CO_2_-exposed oysters (Table II; unpaired t-test, P = 0.07). Long-term hypercapnia also affected the body condition index of oysters that decreased marginally by ~20%, from 3.5 ± 0.6 (control) to 2.7 ± 0.7 (CO_2_-exposed group)(unpaired t-test, N = 5–6, P = 0.052).

**Figure 1:**
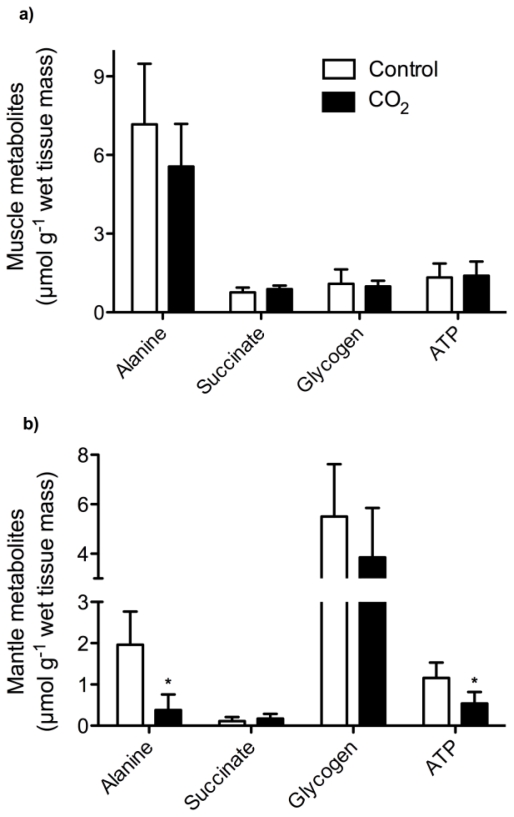

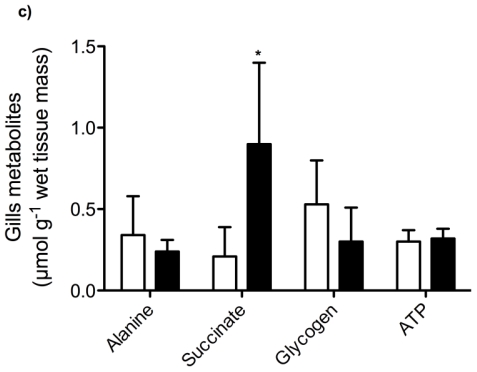
Levels of tissue metabolites in (a) muscle, (b) mantle and (c) gills of control (normocapnia, seawater *P*co_2_ ~ 0.054 kPa) and CO_2_-exposed (hypercapnia, seawater *P*co_2_ ~ 0.15 kPa) oysters, *C. gigas* after long-term incubation at 15 °C. Data are means ± SD with N = 6–8 (control) and N = 6–9 (CO_2_-incubation). * indicates significant differences between control and CO2-incubated animals (unpaired t-test, P < 0.05).

The ^1^H-NMR spectra of the different tissues showed clear and reproducible specific metabolic profiles. Most of the signals did not change significantly between control and CO_2_ exposed oysters in the various tissues, whereas concentrations of some metabolic intermediates were strongly affected by long-term hypercapnia in mantle and gill tissue (Figures 1b, c). In comparison the metabolic profile of muscle tissue was unaffected (Figure 1a). A strong depletion in alanine and ATP levels and a slight (non-significant) decline in glycogen concentrations was found in the mantle of CO_2_-exposed oysters (Figure 1b). In gills, succinate levels were significantly elevated in CO_2_-exposed oysters compared to their control counterparts (Figure 1c). A significant increase in succinate concentrations was also found in hepatopancreas of CO_2_-exposed oysters compared to controls (data not shown).

**Figure 2:**
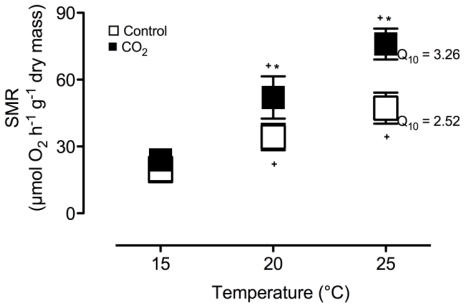
Normalized standard metabolic rate (SMR) in control (normocapnia, seawater *P*co_2_ ~ 0.054 kPa) and CO_2_-exposed (hypercapnia, seawater *P*co_2_ ~ 0.15 kPa) oysters, *C. gigas* during acute warming (5 °C/48 h). Data are means ± SD, N = 6 in each group. + indicates significant differences to the respective data at 15 °C (repeated measures ANOVA, P < 0.02). * indicates significant differences between control and CO_2_-incubated animals at the respective temperature (unpaired t-test, P < 0.05). Differences in Q_10_ values between the groups were marginally significant (unpaired t-test, P = 0.06).

SMR was similar in control and CO_2_-exposed animals when measured at the acclimation temperature of 15 °C (Figure 2). With warming SMR rose significantly in both groups revealing a stronger rise in SMR of CO_2_-exposed compared to control animals as shown by higher Q_10_ values in the former group (Figure 2). As a result, SMR of CO_2_-exposed oysters was significantly higher than in the controls at 20 °C and especially at 25 °C.

**Figure 3:**
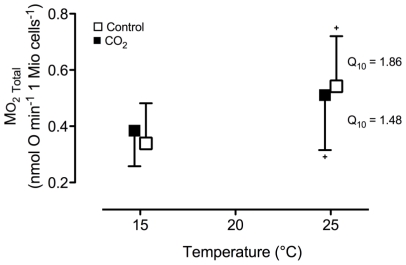
Temperature-dependent respiration rates of isolated gill cells at the respective *in vivo* hemolymph pH of control (normocapnia, seawater *P*co_2_ ~ 0.054 kPa) and CO_2_-exposed (hypercapnia, seawater *P*co_2_ ~ 0.15 kPa) oysters, *C. gigas* after long-term incubation at 15 °C. Data are means ± SD with N = 6 (control) and N = 6–8 (CO_2_-incubation). + indicates significant differences to respective data at 15 °C (paired t-test, P < 0.03). Q_10_ values did not differ significantly between the groups (unpaired t-test, P = 0.383).

Contrary to whole animal SMR, we found no temperature-dependent effect of hypercapnia on respiration rates of isolated gill cells (Figure 3). Cellular respiration was significantly higher at 25 °C compared to 15 °C but the increase was similar in cells isolated from control and CO_2_-exposed animals. Ouabain-sensitive respiration indicating energy demand for ion regulation via Na^+^/K^+^-ATPase comprised 33–37% of the total cellular oxygen demand and was not changed by temperature or by long-term hypercapnia (P > 0.05, data not shown).

Environmental change can be considered stressful if an organism needs to increase energy expenditure on maintenance, defense or repair. Stress-induced metabolic adjustments are aimed at reinstating the metabolic balance (*i.e.*, energy homeostasis) and thus ensuring survival of the individual and, most importantly, of the population. Energy homeostasis implies that the energy demand is covered by sufficient energy supply. Furthermore, there is a net energy gain to invest into production (somatic growth and reproduction). Therefore, any environmental disturbance that reduces this energy investment into production will have direct consequences for the organism’s fitness.

Earlier studies in calcifying marine invertebrates including mollusks indicate that biomineralization is an energetically costly process. In molluskan shells consisting of inorganic crystals (calcite and/or aragonite) and an organic (mostly proteinaceous) matrix [72], production of the organic matrix was proposed to be the main cost-intensive process in shell growth [73]. Compared to the estimated total cost for inorganic shell material (1–2 J·mg^−1^ CaCO_3_), total costs for protein synthesis for shell formation are much higher (29 J·mg^−1^) and may explain the observed inverse relationship between rates of calcification and proportion of organic matrix in shells of marine mollusks [73,74]. However, this is a conservative estimate of the cost of the inorganic shell material in mollusks because it does not take into account such energy-dependent aspects of calcification as the production of enzymes involved in calcium carbonate deposition (e.g., carbonic anhydrase), transport of CaCO_3_ crystals by hemocytes and acid-base regulation at the site of CaCO_3_ deposition [75–77]. Even with this conservative estimate, energetic costs for calcification could account for 75% of the total energy needed for somatic growth and may be up to four times higher than the amount of energy invested in reproduction, as shown in a rocky shore archeogastropod, *Tegula funebralis* [74]. After the shell is deposited, mollusks spend additional energy on its maintenance in order to counteract dissolution and erosion; in some intertidal limpets of the genus *Patella* the annual costs of shell erosion accounted for 8–20% of the total energy invested in production (somatic and shell growth and gonadal output)[78]. Such high energy expenditure for shell deposition and maintenance makes it likely that energetic trade-offs can also occur between shell, somatic growth and reproduction.

Exposure to elevated CO_2_ levels and associated acidification of seawater can inhibit biomineralization rates and increase shell dissolution in marine mollusks [38,40,41,79] or as shown in some species of mollusks, crustaceans and echinoderms, calcification rates increased with decreasing seawater pH up to a certain point [41,51,80]. This may result in elevated metabolic costs of shell deposition and maintenance. The present investigation did not reveal an increase in basal metabolism in OA-exposed oysters when measured at acclimation temperature suggesting: (i) no OA-induced rise in costs or (ii) a shift in energy budget between tissues where a cost depression in one tissue is compensated for by the cost increment in another (see below). In the brittlestar *Amphiura filiformis* [80] the OA-induced increase in calcification rates coincided with an increase in respiration rates and was accompanied by a decrease in muscle mass [80]. The authors interpreted the loss in muscle mass as an energy source for the OA-induced elevated energy demands indicating a trade-off between structure (morphological integrity) and function (arm movement). In contrast, OA-exposed cephalopods *S. officinalis* gained soft body mass at similar rates as control animals although calcification rates were significantly higher in OA-exposed than in control animals [43]. In a following study the authors noted a reduced incorporation of organic matrix in the calcified structure of OA-exposed cephalopods [51] indicating again that a compensatory increase in calcification rates with increasing acidification comes at a cost.

Similar to previous reports on mussels [81] mild CO_2_ accumulation according to OA scenarios had no impact on SMR of oysters at the acclimation temperature (15 °C) indicating neither elevated energy demand nor metabolic rate depression in CO_2_-exposed animals. However, SMR of CO_2_-exposed animals was significantly above that of the normocapnic controls during acute warming indicating that hypercapnia resulted in elevated energy demand when combined with temperature stress. Moreover, the lowered condition index of CO_2_-exposed animals suggested reduced growth efficiency due to a likely shift in energy budget in OA-exposed animals (see discussion above). Furthermore, it has been shown that hypercapnia interferes with protein turnover resulting in lowered protein synthesis in marine organisms [80,82,83], which may explain reduced growth under elevated CO_2_ levels despite unchanged respiration rates.

Whole animal respiration rates gives an estimation of the overall sum of all energy consuming processes, making it likely to oversee small changes in specific processes or if the change in one process is compensated for by another. In line with observations cited above the rise in calcification costs may compensate for metabolic depression in other tissues like muscle or gills such that a net effect of OA on SMR of the whole animal does not occur. In this context, OA-induced alterations in metabolite profiles of oyster gills and mantle indicate that hypercapnia has an impact on metabolic pathways in these tissues. In gills and hepatopancreas, the most notable alteration was an increase in succinate concentration during prolonged exposure to elevated CO_2_ levels. Succinate accumulation is considered an indicator of anaerobic metabolism in bivalves including oysters [84,85]. Succinate accumulation in CO_2_-exposed oysters in our study may only be due to a transition to partial anaerobiosis in the respective organs. We cannot exclude such oxygen limitation to set in as we found a small reduction in hemolymph oxygenation at constant SMR during long-term hypercapnia at 15 °C. This indicates that tissues may not become fully hypoxemic under these conditions but tissue-specific microperfusion may have been disturbed. However, further study is needed to corroborate our conclusions. In contrast to familiar patterns of anaerobic metabolism, no alanine accumulation was observed in gills or hepatopancreas (data for hepatopancreas not shown). Alanine is an early indicator of acute anaerobiosis in marine bivalves including oysters and its accumulation typically precedes that of succinate [84–86]. Hypercapnia also resulted in a strong (by ~80%) depletion of alanine levels in the mantle tissues of oysters. Given that osmotic and hypoxic stress, two major modulators of amino acid levels in bivalves, can be ruled out in our study, the drop in alanine levels of mantle tissue of OA exposed oysters may reflect increased gluconeogenesis. In fully aerobic cells this process may occur and compensate for the putative accumulation of alanine in anaerobic cells. An OA-induced shift in metabolic pathways favoring gluconeogenesis due to an OA-induced impairment of glycolysis was also suggested in fish [87]. We suggest that alanine is transaminated to pyruvate, which together with ATP will be used to build up phosphoenolpyruvate (PEP), with PEP as substrate entering the gluconeogenetic pathway. The glucose synthesized from alanine in the mantle can then be used to fuel the metabolic demand of mantle and most likely transported to other tissues like the gills. However, a nonsignificant trend (due to the high variability between individuals) for a decrease in the glycogen level was observed in the mantle tissue of hypercapnic oysters, which would be consistent with elevated glycolytic flux. Hypoxemia may prevent enhanced gluconeogenesis to be effective in replenishing glycogen levels. Further investigations are needed to determine activities of enzymes involved in glycolysis and gluconeogenesis and/or metabolite fluxes in different tissues to fully unravel the mechanisms of the observed metabolite shifts and their physiological consequences at the wholeorganism level.

Extracellular pH has been identified as an important determinant of metabolic rate of muscle tissue and (vertebrate) liver, and metabolic rate can drop if pH_e_ decreases below a certain threshold level [33]. OA exposure caused a marked acidosis in extracellular fluids of *C. gigas*, which remained largely uncompensated as bicarbonate levels increased only slightly. Along with the relative stability of bicarbonate levels, extracellular [Ca^2+^] also did not change during hypercapnia in *C. gigas* indicating no dissolution of shell or tissue calcium carbonate. In contrast, hypercapnia-induced acidification of seawater (pH = 7.3) resulted in a decrease of pH_e_ in *M. galloprovincialis* and a significant increase in hemolymph Ca^2+^ levels which was interpreted as partial buffering of pH_e_ by shell dissolution [32]. Our findings are in line with recent studies in blue mussels *M. edulis* and the Greenland smoothcockle, *Serripes groenlandicus*, where no significant change in extracellular [HCO_3_ ^−^] and [Ca^2+^] was noted with increasing seawater *P*co_2_ up to 0.4 kPa [88–90]. Possibly, the degree of involvement of the shell and tissue CaCO_3_ in buffering of the hemolymph pH depends on the degree of acidification and may become notable when the environmental pH decreases below a certain species-specific threshold. Furthermore, as discussed by Hardewig *et al.* [91] “metabolism itself may contribute to the regulation of acid-base balance”. Pörtner [92] suggested that PEP can be carboxylated under hypercapnia resulting in an increase of succinate (as observed in this study) and propionate concentrations accompanied with the consumption of bicarbonate or CO_2_, an additional benefit under hypercapnic conditions [88].

Concomitant with the more acidic pH_e_, Michaelidis *et al.* [32] found reduced respiration rates in OA-exposed mussels. Also in oysters (*C. virginica*), Willson and Burnett [69] reported a lowered oxygen uptake in animals subjected to high CO_2_ partial pressures: high (0.8–1 kPa *P*co_2_, seawater pH ≤ 7) compared to controls at low (<0.1 kPa *P*co_2_, seawater pH 8.2). In line with findings at the whole animal level, oxygen uptake of isolated gill tissue from oysters, *C. virginica* was depressed under the low pH conditions irrespective of the CO_2_ levels [69]. Reipschläger and Pörtner [68] and Pörtner *et al.* [93] provided conclusive evidence that and how metabolic depression in isolated body wall musculature of the peanut worm *Sipinculus nudus* under hypercapnia was specifically due to lowered extracellular pH rather than intracellular pH, elevated *P*co_2_ or modified bicarbonate levels. In our experiments, seawater pH fell to 7.7 and no pH_e_ compensation was observed (pH_e_ = 7.1). At the temperatures tested CO_2_-exposed animals showed no sign of metabolic depression and, similarily, isolated gill cells showed no change in respiration rates when measured in buffer that was adjusted to the respective pH_e_ [7.6 (control) *vs.* 7.1 (CO_2_-group)]. Different temperature sensitivities suggest, however, that upon cooling, CO_2_ may cause metabolic rate to fall below controls. Our findings suggest that within the tested temperature range the pH_e_ reached during exposure to mild hypercapnia as in this study is found above the threshold triggering overall metabolic rate depression in *C. gigas*.

Ion regulation capacity via Na^+^K^+^-ATPase showed no difference between gill cells isolated from control and CO_2_-exposed animals. The data for Na^+^K^+^-ATPase regulation under hypercapnia are sparse and contradictory suggesting species-specific thresholds in CO_2_-induced impact on Na^+^K^+^-ATPase capacity. Thus, in some species elevated CO_2_ levels have no effect on activity of Na^+^K^+^-ATPase (2 kPa *P*co_2_, [94]) whereas in others increased Na^+^K^+^-ATPase activity was observed after acclimation to long-term hypercapnia (1–5 kPa *P*co_2_, [17,95]). Melzner *et al.* [3] observed a concentration-dependent effect of CO_2_ on Na^+^K^+^-ATPase activity in cod gills with unchanged activity at 0.3 kPa *P*co_2_, and increased activity at 0.6 kPa *P*co_2_. In *S. nudus*, extracellular pH modulates the energy costs for acid-base regulation by a shift from less to more ATP-efficient ion transporters during hypercapnia resulting in a reduction of Na^+^K^+^-ATPase activity due to reduced requirement for sodium regulation [68,93,96]. In CO_2_-exposed oysters the observed lowering of extracellular [Na^+^] and elevation of [K^+^] compared to controls is consistent with the hypothesized shift to more ATP-efficient ion transporters. If it holds true in oysters, the associated changes in Na^+^K^+^-ATPase activity must be small and undetectable by the method employed in our present study (determination of ouabainsensitive respiration). To date, many different ion transporters have been identified [97,98] but the complex interplay of these proteins regulation ion- and pH balance is not yet fully understood and requires further investigation.

## 3. Experimental Section

### 3.1. Animal collection and maintenance

Wild adult oysters, *Crassostrea gigas* (80 to 130 mm shell length) were collected at the North Sea coast near the islands of Langeoog and Baltrum (catch position: 53°42’ North, 7°26’ East) in March 2009. Salinity was ~29 psu and water temperature ~7 °C at the time of collection. Animals were covered with wet towels in polystyrene boxes at 8–12 °C and transported to the Alfred Wegener Institute (Bremerhaven, Germany) within 24 h of collection. Oysters were separated from each other, washed and cleaned from epibionts and maintained in recirculating aerated water tanks with filtered natural seawater from the North Sea at 15 ± 1 °C and 32 ± 1 psu. Following pre-acclimation for at least 30 days, during which no mortality occurred, oysters were randomly divided into a control group and a CO_2_-exposed group with two to three replicate tanks per group. Animal density in the tanks was maintained at one oyster per 6 L of seawater. Oysters were fed three times a week *ad libitum* with a commercial algal blend containing *Nannochloropsis*, *Phaeodactylum tricornutum* and *Chlorella* (DT’s Live Marine Plankton, Coralsands, Germany, www.coralsands.de).

For CO_2_ incubations, elevated *P*co_2_ was produced by gas mixtures of 99.9% CO_2_-free air and 0.1% CO_2_ gas using Wösthoff gas mixing pumps (Wösthoff GmbH, Germany, http://www.woesthoff.com). All animal tanks (experimental, incubation and reservoir tanks for water changes) were continuously bubbled with the ambient air or air-CO_2_ mixtures as appropriate, and water change was performed every other day to ensure adequate water quality. Parameters were measured in water samples at least three times a week immediately before and 1 to 2 h after a water change (see Table 2).

Alkalinity was measured by potentiometric titration (METROHM Prozessanalytik GmbH&Co, Germany, see [47]). The CO_2_ incubations lasted for 26–55 days and the physicochemical parameters of the seawater are reported in Table I. Parameters of carbonate chemistry were calculated from temperature, salinity, pH and total alkalinity of the seawater with the software CO2SYS [48] using the equilibrium constants of Mehrbach *et al.* [49] as refitted by Dickson and Millero [50–52].

### 3.2. Tissue and hemolymph collection

To minimize stress, oysters were taken out from the incubation tanks one at a time and put on ice for immediate processing. Hemolymph samples were taken anaerobically from the intact pericardium (no hemolymph samples were taken if pericardium was damaged) with gas-tight syringes and either measured immediately (for acid-base parameters and gas concentrations) or prepared for further analyses of ion concentrations. Tissue samples (gills, mantle, muscle, hepatopancreas) were taken, immediately freeze clamped and stored in liquid nitrogen for further analyses.

### 3.3. Determination of hemolymph acid-base parameters, P_e_co_2_, P_e_o_2_ and C_e_co_2_

Hemolymph parameters (pH_e_, *P*_e_co_2_ and *P*_e_o_2_) were measured immediately after sampling using a blood gas analyzing system (combined system with glass pH electrode, *P*co_2_ and *P*o_2_ electrode) from Eschweiler (MT 33, Eschweiler, Germany). The instrument was calibrated at the respective acclimation temperature (15 °C) and allows for accurate measurements at low gas concentrations. Total CO_2_ concentration in hemolymph (C_e_co_2_) was analyzed by gas chromatography (Agilent 6890N GC System, Agilent Technologies, USA, see 53). Apparent bicarbonate concentrations in the hemolymph ([HCO^−^ _3_]_e_) were calculated as follows:

$${\left[{{\text{HCO}}^{-}}_{3}\right]}_{\text{e}}={\text{C}}_{\text{e}}{\text{CO}}_{2}-(\alpha {\text{CO}}_{2}\times P_{\text{e}}{\text{CO}}_{2})$$

where C_e_co_2_ = total CO_2_ concentration in hemolymph (mM), αCO_2_ = solubility of CO_2_ in hemolymph calculated after Heisler [54] (0.0525 mmol L^−1^ Torr^−1^), and *P*_e_co_2_ = partial pressure of CO_2_ in hemolymph (Torr).

For ion analysis (sodium [Na^+^], potassium [K^+^], magnesium [Mg^2+^], calcium [Ca^2+^] and ammonium [NH^4 +^]), hemolymph samples were centrifuged (19,000 g, 20 min, RT) and plasma samples were stored at −80 °C for later measurements. Defrosted plasma samples were diluted 1:300 to 1:500 with deionised H_2_O which yielded good signal to noise ratios, and ion concentrations were measured via ion chromatography (ICS-2000, Dionex®, Germany, see [55]).

### 3.4. Determination of metabolites

**Figure 4:**
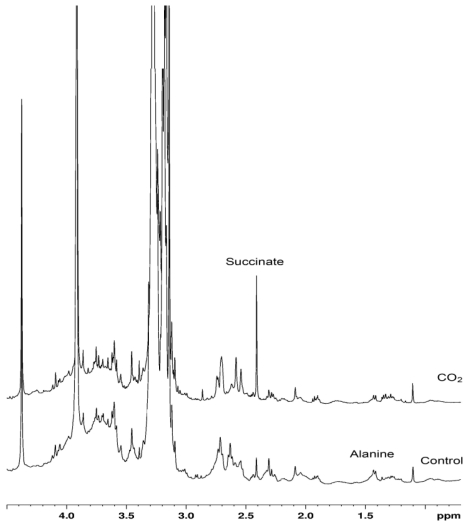
Example of ^1^H-NMR spectra from gill tissue extracts of control and CO_2_-exposed oysters. A clear increase in succinate can be observed following hypercapnia.

For the determination of metabolite concentrations in muscle, mantle and gill tissues, frozen tissue samples were powdered with mortar and pestle under liquid nitrogen. Tissue powder (~0.3 g) was added to an excess (5×) volume of ice-cold 0.6 M perchloric acid (PCA) and homogenized by ultrasonic treatment (0 °C, 360 Watt). Precipitated protein was removed by centrifugation (0 °C, 2 min at 16,000 g). The supernatant was neutralized with 5 M potassium hydroxide (KOH) to pH 7.0–7.5. Precipitated potassium perchloride was removed by a second centrifugation (0 °C, 2 min at 16,000 g). Extracts were stored at −80 °C for further analyses. For NMR spectroscopy, samples were freeze-dried by centrifugation in a SpeedVac. Dried samples were diluted in D_2_O (resulting in a final concentration of 0.3 g initial tissue powder per mL), mixed and transferred to 5 mm NMR tubes. Fully relaxed one-dimensional (1D), one pulse ^1^H-NMR spectra of tissue extracts were recorded with an inversed ^1^H-broad band probe (^1^H/BBI) using a 9.4 T Avance 400 WB spectrometer (Bruker Biospin GmbH, Germany). Parameters were as follows: flip angle 90°, spectral width 4k, time domain 16k, acquisition time 2.04 s, relaxation delay 12 s, 64 scans (+2 dummy scans), resulting in a scan time of 15.3 min. Spectra were processed and analyzed using TopSpin 2.5 (Bruker Biospin GmbH, Germany). Prior to Fourier transformation all data were zero filled to 64k and processed with an exponential multiplication of 0.5 Hz. After phase and baseline correction, spectra were calibrated to TMS (at 0.0 ppm) that was added to the tissue extracts. Specific metabolites were identified using chemical shift tables [56–62]. Following signals were identified in the different tissues: formate, histidine, phenylalanine, tryptophan, ATP, glycogen, glycine-betaine, betaine/TMAO, choline, arginine, aspartate, ketoglutarate, succinate, glutamine, GABA, acetate, alanine, propanediol. Metabolites of interest such as succinate/alanine were analysed in more detail (see Figure 4 and the result part). Metabolite concentrations were determined by integration of NMR signals using the integration routine in TopSpin. All integrals were calibrated to the integral of a 1mM succinate solution that was determined in a separate standard sample using the same acquisition parameters as for our tissue samples.

### 3.5. Determination of standard metabolic rate and animal condition index

SMR was measured as resting oxygen consumption in control and CO_2_-exposed oysters using microfiber optic oxygen probes (Tx-Type, PreSens GmbH, Germany, http://www.presens.de)[63]. To avoid interference with postprandial metabolism and feces excretion, animals were fasted for 24 h prior to the start of SMR recordings. Two-point calibration (N_2_-bubbled seawater for 0% and air-bubbled seawater for 100% air saturation) was performed at each temperature (15, 20 and 25 °C). The oxygen consumption rate was first measured at the acclimation temperature of 15 °C, then the temperature was increased to 20 °C overnight and oxygen consumption measured the following day. After 48h the temperature was increased again by 5 °C overnight and the final determination of oxygen consumption was conducted at 25 °C. After measurements, oysters were dissected and tissue dry mass was determined. SMR was calculated as follows:

$$\text{SMR}=(\Delta P{\text{O}}_{2}\times \beta {\text{O}}_{2}\times \text{Vfl})/{\text{M}}^{0.8}$$

where SMR is the oxygen consumption normalized to 1 g dry tissue mass (μmolO_2_ g^−1^ dry mass h^−1^), Δ *P*o_2_ the difference in partial pressure between in- and out-flowing water (kPa), βO_2_ the oxygen capacity of water (μmolO_2_ L^−1^ kPa^−1^), Vfl the flow rate (L h^−1^), M the oyster tissue dry mass (g) and 0.8 is the allometric coefficient for *C. gigas* [64].

A general condition index (CI) of the experimental oysters was calculated as follows [63,65,66]:

CI=tissue dry mass (g)/shell dry mass (g)×100

### 3.6. Determination of cellular respiration rates and fractional cost for ion regulation via Na^+^/K^+^ ATPase

Cells were isolated from gills of control and CO_2_-exposed oysters using a protocol modified from Cherkasov *et al.* [67]. Gills from 2–3 oysters were pooled, minced and washed twice in ice-cold buffer A (in mM: NaCl 423, KCl 9.1, CaCl_2_ 9.3, NaHCO_3_ 2.1, Hepes 30 at pH 7.5 or 7.1 for cell isolation from control or CO_2_-incubated oysters, respectively). The tissue was digested with 0.125% Trypsin/EDTA in balanced Hank’s solution adjusted to 720 mOsm with sucrose. Dissected gills were gently shaken in the digestion solution for ~30 min at room temperature (0.5 g tissue mL^−1^ solution). Digestion was stopped by adding 5–10% (v:v) fetal calf serum. The extract was filtered through 100 μm sterile nylon mesh, washed twice with buffer A and centrifuged for 10 min at 400g and 4 °C to pellet the cells. Cell pellets were washed three times in buffer A, combined and finally resuspended in buffer B (in mM: NaCl 423, KCl 9.1, CaCl_2_ 9.3, NaHCO_3_ 2.1, MgCl_2_ 22.9, MgSO_4_ 25.5, glucose 15, Hepes 30 at pH 7.5 or 7.1 for cell isolation from control or CO_2_-incubated oysters, respectively). In the pilot experiments, we measured cellular respiration using buffer B adjusted for pH and *P*co_2_ to mimic the hemolymph parameters of control and CO_2_-exposed oysters, respectively. The elevated *p*CO_2_ of the buffer had no effect on cell respiration (data not shown) which agrees with the results of previous studies [68,69]; therefore, in the subsequent experiments, only pH was adjusted to simplify the experimental logistics.

Cell density was determined in a Fuchs–Rosenthal counting chamber and adjusted to 10 × 10^6^ cells mL^−1^. Cell viability was assessed by using a standard Trypan Blue exclusion assay and was found to be >80%. Cellular respiration was determined in 1 mL water-jacketed, air-tight chambers using microfiber optic oxygen probes (Tx-Type, PreSens GmbH, Germany, http://www.presens.de). A two-point calibration was performed at each temperature (15 and 25 °C) using saturated sodium sulfide solution for 0% and air-bubbled medium for 100% air saturation. Cellular oxygen consumption in the absence of inhibitors (MO_2 total_), and MO_2_ in the presence of 1mM and 10mM ouabain to inhibit Na^+^/K^+^-ATPase were recorded (MO_2 ouabain_, both concentrations showed a similar degree of inhibition of cellular MO_2_ and, thus, were counted as replicates)[70,71]. Cell respiration rates were calculated as follows:

$${\text{MO}}_{2}(\text{nmol O }{\text{min}}^{-1}10^{6}\;{\text{cells}}^{-1})=-\text{m}\times (P{\text{O}}_{2}/100\%)\times \beta {\text{O}}_{2}\times {\text{V}}_{\text{cell}}/\text{N}$$

where m is the slope of the decrease of oxygen saturation in the respiration chamber (% min^−1^), *P*o_2_ is the oxygen partial pressure at 100% saturation (Torr), βo_2_ is the oxygen capacity of the solution (μmol O_2_ L^−1^ Torr^−1^), V_cell_ is the volume of the cell suspension used (L), and N is the number of cells in the V_cell_ of cell suspension.

Oxygen demand for ion regulation via Na^+^/K^+^-ATPase was calculated as follows:

$${\text{MO}}_{2\text{NaK ATPase}}(\text{nmol O }{\text{min}}^{-1}10^{6}\;{\text{cells}}^{-1})={\text{MO}}_{2\;\text{total}}-{\text{MO}}_{2\text{ouabain}}$$

Prior to oxygen consumption measurements 0.5–1 mL of cell suspension was centrifuged and 2/3 of the supernatant was exchanged by fresh buffer B to ensure saturated oxygen and substrate concentration for maximal cell respiration. All chemicals and media were obtained from Sigma- Aldrich (Germany).

### 3.7. Statistical analysis

Statistical analyses were carried out using InStat 3.0b and Prism 5.0b (GraphPad Software, Inc.). Differences between measured parameters of control and CO_2_-incubated animals were determined by unpaired t-test (parametric test) or Mann-Whitney test (non-parametric test) as appropriate depending on the normality of distribution of the dependent variable. Temperature effects on whole animal and cellular respiration rates were tested by repeated measures ANOVA and paired t-test, respectively. The differences were considered significant if P < 0.05 unless stated otherwise. All data are presented as mean values ± standard deviation (SD).

## 4. Conclusions

Our present study demonstrates that CO_2_ levels corresponding to expected OA scenarios are likely to interfere with the energy metabolism of oysters. This may reflect vulnerability to OA and temperature extremes. These findings are especially noteworthy because oysters, like other estuarine invertebrates, are normally exposed to broad fluctuations in CO_2_ levels, pH and temperature in their habitats and thus should be better adapted to these changes than their deep-water or open-ocean counterparts. Nevertheless, chronic hypercapnia affects energy metabolism even in this eurybiont species especially when combined with elevated temperature. Synergistic effects of elevated temperature and hypercapnia were also identified in other bivalves [89,99] and are consistent with earlier reports that elevated temperature enhanced the sensitivity to a variety of environmental stressors such as pollution, hypercapnia or oxygen deficiency [1,4,100]. However, species-specific physiological differences in biomineralization as well as energy metabolism and acid-base regulation may shape differential sensitivities of various marine invertebrates to OA and elevated temperature thus complicating the picture of ecosystem-level responses of marine organisms to a high CO_2_ world. Further studies are critically needed to determine the range of sensitivities of key marine species to OA and global climate change and the mechanisms setting limits to their tolerance to elevated temperatures and low pH in the future oceans. Analyses of energy metabolism as in the present study can provide a useful integrative view of stress effects on physiological performance of a variety of marine species and characterize their tolerance and tolerance limits in the face of global change.

## Figures and Tables

**Table 1:** Hemolymph parameters of oyster, *C. gigas* after long-term incubation at control (normocapnia, seawater *P*co_2_ ~ 0.054 kPa) and elevated CO_2_-concentrations (hypercapnia, seawater *P*co_2_ ~ 0.15 kPa, 26 to 55 days) at 15 °C.

Parameter/Group	Control	CO_2_-incubation

pH_e_	7.60 ± 0.10	7.09 ± 0.18*
*P*_e_CO_2_ (kPa)	0.15 ± 0.04	0.54 ± 0.19*
*P*_e_O_2_ (kPa)	11.44 ± 3.67	9.43 ± 2.29°
C_e_CO_2_ (mM)	1.67 ± 0.11	2.15 ± 0.30*
HCO^−^_3_ (mM)	1.28 ± 0.11	1.78 ± 0.29*
Ca^2+^ (mM)	7.2 ± 0.6	6.2 ± 0.1°
Na^+^ (mM)	445.4 ± 16.0	422.3 ± 7.4*
K^+^ (mM)	11.9 ± 0.8	13.0 ± 0.9*

(pH_e_: extracellular pH; C_e_CO_2_: total dissolved inorganic carbon content). Data are means ± SD with N = 7–16 (control) and N = 6–23 (CO_2_-incubation).

* indicates significant differences between control and CO_2_-incubated oysters (unpaired t-test, P < 0.05).

° indicates marginally significant differences between control and CO_2_-incubated oysters (unpaired t-test, P = 0.07).

**Table 2:** Physicochemical conditions of seawater during control (normocapnia) and CO_2_-incubation (hypercapnia) of oyster, *C. gigas* at 15 °C.

Parameter/Group	Control	CO_2_-incubation

Salinity (psu)	32.1 ± 0.5	31.3 ± 0.4
pH _NBS_	8.07 ± 0.04	7.68 ± 0.07
*P*CO_2_ (kPa)	0.059 ± 0.008	0.15 ± 0.026
[HCO^−^_3_] (mmol kg^−1^)	2.09 ± 0.09	2.24 ± 0.12
Ω Ar	2.31 ± 0.30	0.87 ± 0.15
Ω Ca	3.59 ± 0.44	1.36 ± 0.24

NBS: National Bureau of Standards; *P*CO_2_: seawater partial pressure of CO_2_; Ω Ar and Ca: saturation state of aragonite and calcite, respectively. Data are means ± SD with N = 11 (control) and N = 37 (CO_2_-incubation).
